# *Prdm14* promotes germline fate and naive pluripotency by repressing FGF signalling and DNA methylation

**DOI:** 10.1038/embor.2013.67

**Published:** 2013-05-14

**Authors:** Nils Grabole, Julia Tischler, Jamie A Hackett, Shinseog Kim, Fuchou Tang, Harry G Leitch, Erna Magnúsdóttir, M Azim Surani

**Affiliations:** 1 Wellcome Trust/Cancer Research UK Gurdon Institute, Department of Physiology, Development and Neuroscience, and Wellcome Trust Medical Research Council Stem Cell Institute, University of Cambridge, Tennis Court Road, Cambridge CB2 1QN, UK

**Keywords:** DNA methylation, FGF signalling, pluripotency, *Prdm14*, primordial germ cells

## Abstract

Primordial germ cells (PGCs) and somatic cells originate from postimplantation epiblast cells in mice. As pluripotency is lost upon differentiation of somatic lineages, a naive epigenome and the pluripotency network are re-established during PGC development. Here we demonstrate that *Prdm14* contributes not only to PGC specification, but also to naive pluripotency in embryonic stem (ES) cells by repressing the DNA methylation machinery and fibroblast growth factor (FGF) signalling. This indicates a critical role for *Prdm14* in programming PGCs and promoting pluripotency in ES cells.

## INTRODUCTION

Primordial germ cell (PGC) specification in mice commences in the proximal epiblast cells in response to BMP4 signalling at embryonic day (E) 6.25 just before the onset of gastrulation. By E7.25, approximately 30–40 founder PGCs are established following expression of the three key regulators: *Prdm1* (BLIMP1), *Prdm14* and *Tcfap2c* (AP2γ) [1–3]. The segregation of PGCs from neighbouring mesoderm progenitor cells entails repression and reversal of the initiation of the somatic programme, and re-establishment of the pluripotency network in conjunction with changes in chromatin modifications [4, 5].

Expression of *Prdm14* is confined to PGCs and pluripotent cells only, where it has a critical role for the regulation of pluripotency genes, and it promotes resetting of the epigenome [3, 6]. *Prdm14*-deficient PGCs are specified, but fail to proliferate and are eventually lost during migration towards the genital ridges. The mutant PGCs exhibit diminished expression of *Sox2* and *Stella*, and fail to show global histone methylation changes as observed in wild-type PGCs [5]. *Prdm14* promotes a naive pluripotent state in differentiation-primed epiblast stem cells [6], while loss of *Prdm14* in embryonic stem (ES) cells induces primitive endoderm (PE) fate [7]. *PRDM14* is equally important for preventing differentiation of human ES cells, and can enhance somatic cell reprogramming [8, 9].

Here we explored the role of *Prdm14* in PGCs and in mouse ES cells. We find that *Prdm14* reverses and protects cells from acquiring somatic fates partly by attenuating mitogen-activated protein kinase (MAPK) signalling, thereby stabilizing a naive pluripotent state. Furthermore, *Prdm14* represses the DNA methyltransferase machinery, further promoting naive pluripotency.

## RESULTS AND DISCUSSION

### Loss of PGC-specific gene expression

**Figure 1:**
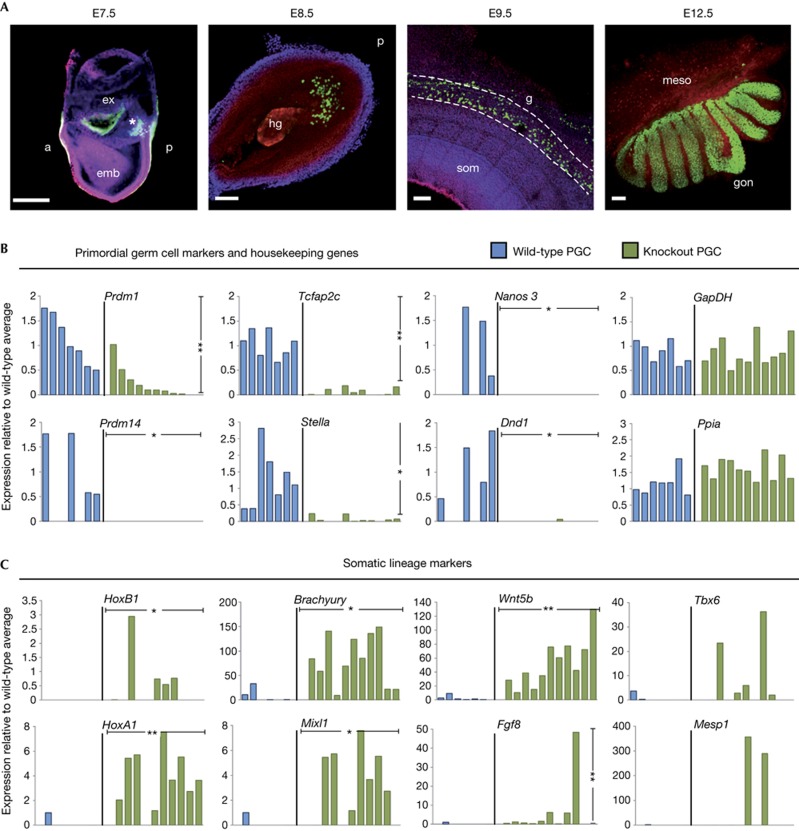
Loss of germline-specific expression and gain of somatic fate markers in *Prdm14* mutant PGCs. (**A**) Stages of wild-type PGC development (PGCs marked by GFP in green). The founder population of PGCs forms a cluster (E7.5; *Prdm1*-GFP, marked by asterisk), enters the hindgut region (E8.5; *Stella*-GFP), migrates through the gut (E9.5; *Oct4*-ΔPE-GFP) and enters into the gonads (E12.5; *Oct4*-ΔPE-GFP) to continue their development into gametes. (scale bars=100 μm). (**B**, **C**) Gene expression levels relative to wild-type average, which is set to 1 (where expression was absent in all wild-type samples, expression levels are relative to knockout average) of individual wild-type (blue) or knockout (green) PGCs. Single cells were ordered according to levels of *Prdm1* expression, with values normalized with *Arbp*. Transcript levels are shown for PGC genes and two housekeeping genes (*Gapdh* and *Ppia*; **B**) and somatic lineage markers (**C**). Welch’s *t*-test was used to calculate statistical significances of differences in expression levels (vertical bars) between wild-type and mutant PGCs. The χ^2^ test was used to determine statistical significances for expression frequency differences (horizontal bars). (*P<0.05; **P<0.01). a, anterior; E, embryonic day, emb, embryonic and ex, extra-embryonic regions; GFP, green fluorescent protein; hg, hindgut; g, gut; som, somites; meso, mesonephros; gon, gonad; PE, primitive endoderm; p, posterior; PGC, primordial germ cells.

To investigate the consequences of loss of *Prdm14* in the germline, we generated *Prdm14*-null mice (supplementary Fig S1A–C online), and examined gene expression in single-mutant PGCs. While development of wild-type PGCs follows an orderly expression of *Prdm1* (BLIMP1), *Stella*, *Tcfap2c*, *Nanos3* and *Dnd1* (Fig 1A,B), *Prdm14*-null PGCs showed diminished expression of these genes (Fig 1B; supplementary Fig S1D,E online), as well as of regulators of pluripotency (supplementary Fig S1F).

Interestingly, genes associated with a somatic fate, such as *HoxB1* and *HoxA1*, were derepressed in mutant PGCs at E8.5 (Fig 1C). Mutant PGCs showed, in particular, strong expression of primitive streak genes like *Brachyury* and *Mixl1*, which is in line with their shared developmental history with the neighbouring mesodermal cells. Notably, *Wnt5b* is strongly induced in mutant cells (Fig 1C), which is consistent with the location of PGCs posteriorly to the primitive streak. However, we did not observe upregulation of extra-embryonic endoderm genes in mutant cells (supplementary Fig S1G online), despite previous reports that *Prdm14* represses them in ES cells [6].

Together, these results demonstrate that loss of *Prdm14* causes loss of PGC identity by E8.5, which was less evident in the previous analysis at E7.5 [3]. Most notably, mutant cells acquire gene expression that is characteristic of adjacent somatic cells, indicating that *Prdm14* is crucial for PGC specification by promoting expression of germ cell genes while repressing somatic genes.

### *Prdm14* modulates FGF signalling and DNA methylation

**Figure 2:**
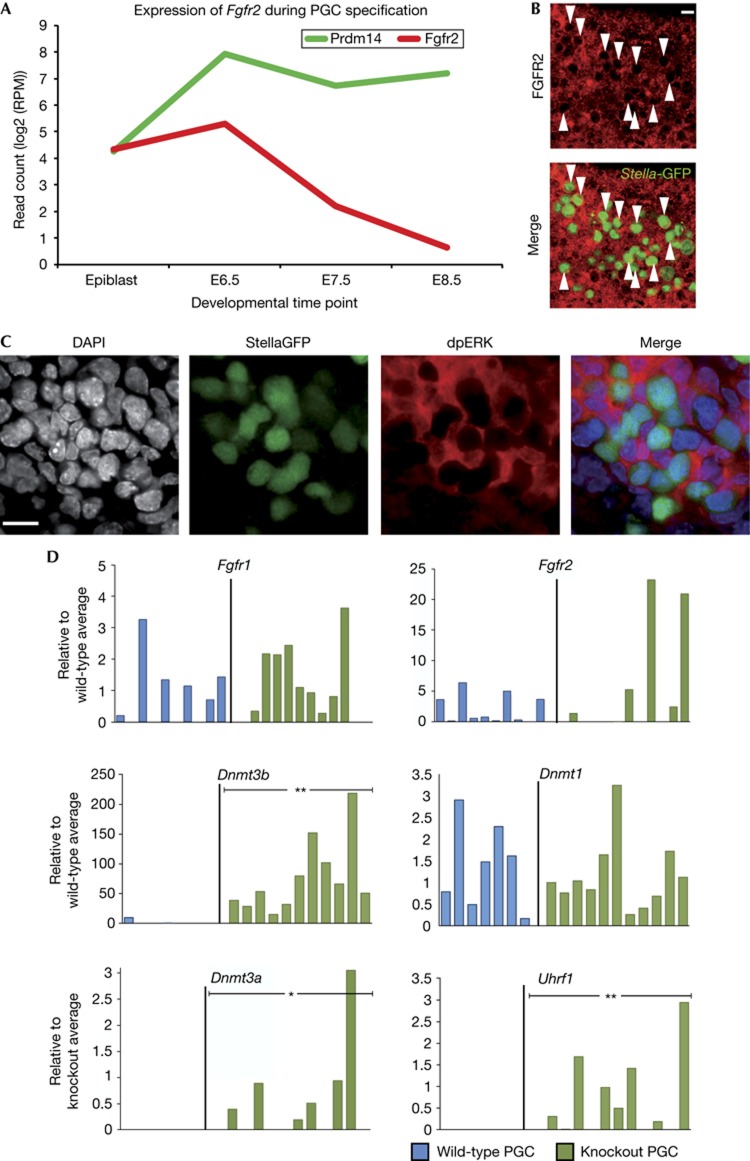
*Prdm14*-deficient PGCs fail to repress *Fgfr2* and DNA methyltransferases. (**A**) Average changes in transcript levels of *Prdm14* and *Fgfr2* over the course of PGC specification determined by single-cell RNA sequencing of two wild-type cells. (**B**) Whole-mount immunostaining for FGFR2 (red) and PGCs, marked by a *Stella*GFP reporter (green, arrowheads) in an E8.5 wild-type embryo (scale bar=15 μm). (**C**) Whole-mount immunostaining for PGCs, marked by *Stella*GFP (green), and phosphorylated ERK (red) in wild-type embryos at E8.75 (scale bar=15 μm). (**D**) Expression of FGF receptors or genes involved in the regulation of DNA methylation in single wild-type (blue) or knockout (green) PGCs. Expression was normalized with *Arbp* and is shown relative to the average wild-type level, which was set to 1 (where expression was absent in all wild-type samples, expression levels are relative to knockout average). Statistical significances by χ^2^ test. (*P<0.05, **P<0.01). DAPI, 4,6-diamidino-2-phenylindole; E, embryonic day; ERK, extracellular signal-regulated kinase; FGF, fibroblast growth factor; GFP, green fluorescent protein; PGC, primordial germ cells.

As initiation of lineage priming and perturbation of the pluripotency network are evoked by FGF signalling in ES cells [10], we examined the status of this pathway in PGCs. Indeed, single-cell transcriptome profiling of wild-type PGCs showed that *Fgfr2* is specifically downregulated at the onset of *Prdm14* expression (Fig 2A), which was confirmed by whole-mount immunostainings for FGFR2 in E8.5 embryos (Fig 2B). Intriguingly, PRDM14 was shown to bind and repress *Fgfr2* in ES cells [7], suggesting a potentially direct regulation in PGCs as well.

We next examined extracellular signal-regulated kinase (ERK) phosphorylation as an indicator of MAPK pathway activity in migratory PGCs and found that while hindgut cells show strong phosphorylation of ERK, there was essentially no ERK phosphorylation in PGCs, further supporting the notion of PRDM14-induced repression of the MAPK pathway (Fig 2C). In addition, some mutant PGCs showed increased levels of *Fgfr2*, indicating that this might be due to the absence of *Prdm14* (Fig 2D). Based on these observations, it is possible that the loss of *Prdm14* causes increased sensitivity to FGF signalling, which could explain changes in gene expression in mutant PGCs and their subsequent elimination.

Development of PGCs is accompanied by the onset of DNA demethylation [5], which in part allows for reversal of the epigenetic silencing of genes at the postimplantation epiblast stage, notably of key germline genes [11–13]. Accordingly, DNA methyltransferases are downregulated in wild-type PGCs [4]. In contrast, *de novo* methyltransferases, *Dnmt3b* in particular, exhibit expression in mutant PGCs (Fig 2D). Also, repression of *Uhrf1*, the essential co-factor for the *Dnmt1* maintenance methyltransferase, observed in wild-type PGCs [4], does not occur in mutant cells. Therefore, *Prdm14* appears to be implicated in the repression of DNA methylation in the germline, which in turn may allow for the expression of germline genes [11], such as *Dazl, Tex19.1, Rhox9* and *Sycp3*. Our analysis suggests that *Prdm14* is involved in the downregulation of *Fgfr2* and repression of ERK activation, as well as the repression of DNA methylation, which together may ensure repression of the somatic programme in PGCs and re-expression of genes of the germ cell lineage.

### Regulation of gene expression in ES cells

**Figure 3:**
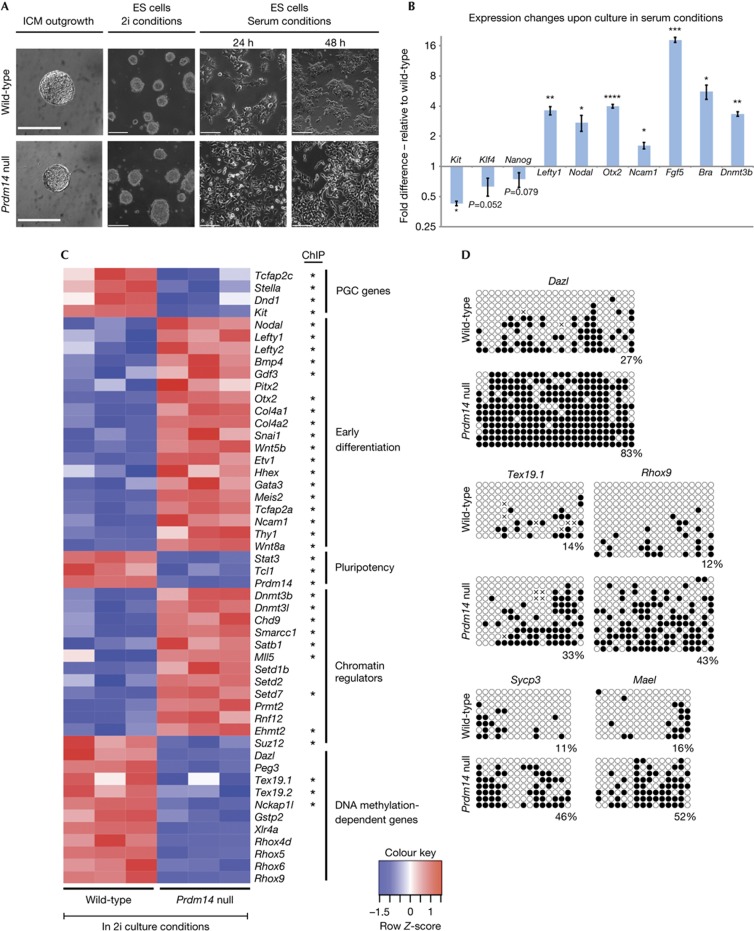
Control of lineage marker expression and DNA methylation by *Prdm14* in ES cells. (**A**) Derivation of *Prdm14*-null ES cells in 2i culture conditions and morphological changes triggered by a shift to serum culture conditions (scale bar=190 μm for ICM outgrowths and 240 μm for all other images). (**B**) Gene expression changes induced by culture in serum conditions for 48 h in *Prdm14*-null ES cells. Values are relative to wild-type cells and normalized to *Gapdh* (*n*=three independent biological repeats). Error bars represent the standard error of the mean (s.e.m.) and statistical significance was assessed by *t*-test (*P⩽0.05; **P⩽0.01; ***P⩽0.001; ****P⩽0.0001) (**C**) Heatmap of genes with significant expression differences (FDR<0.01) between wild-type and *Prdm14*-deficient ES cells in 2i culture conditions. Genes determined to be direct target genes by ChIPseq for PRDM14 in mouse ES cells [7] are highlighted by an asterisk. (**D**) Bisulphite sequencing comparing promoter DNA methylation of DNA methylation-dependent genes in wild-type and *Prdm14*-null ES cells cultured in 2i conditions. ES, embryonic stem; FDR, false discovery rate; ICM, inner cell mass; PGC, primordial germ cell; s.e.m., standard error of the mean.

To further explore the molecular basis of the role of *Prdm14*, we turned to the more tractable ES cells where *Prdm14* plays a role in promoting pluripotency [6–9]. We first derived *Prdm14*-deficient ES cells in 2i culture conditions containing MEK and glycogen synthase kinase-3 inhibitors (Fig 3A) [14, 15]. While their maintenance was not affected by the loss of *Prdm14* in these conditions, transfer to classical culture conditions with serum and leukaemia inhibitory factor (LIF) induced their differentiation (Fig 3A,B), confirming that *Prdm14* has a role in the maintenance of pluripotent ES cells, which is not overtly evident in 2i culture conditions. We therefore sought evidence for the global impact of loss of *Prdm14* in these ES cells by microarray analysis, which indeed revealed significant differences (Fig 3C; supplementary Table S1 online).

First, we found that genes that are essential for early PGC development, such as *Tcfap2c*, *Dnd1* and *Kit* along with the early PGC marker *Stella*, showed reduced expression levels in the absence of *Prdm14*. This is consistent with our findings that *Prdm14*-deficient PGCs lose expression of germ cell genes. Second, we found that *Prdm14*-deficient ES cells in 2i showed higher expression levels of early differentiation markers (Fig 3C, supplementary Fig S2 online). Genes such as *Nodal*, *Lefty1*, *Lefty2* and *Bmp4* that promote developmental progression in the postimplantation epiblast and are usually repressed in ES cells are upregulated in the absence of *Prdm14*. In addition, other differentiation markers that are only expressed during lineage acquisition, like *Wnt5b*, *Snai1, Hhex* and *Ncam1*, were upregulated, which is reminiscent of the upregulation of somatic genes in mutant PGCs.

While the main regulators of pluripotency, such as *Oct4*, *Sox2* and *Nanog*, were not affected, the pluripotency-associated gene *Stat3*, which is crucial for inducing and maintaining naive pluripotency [16], and *Tcl1*, a positive regulator of AKT signalling that promotes proliferation in ES cells and the early embryo [17], were significantly downregulated upon loss of *Prdm14* in ES cells (Fig 3C). Moreover, we found histone H3 lysine-9 methyltransferase *Ehmt2* to be upregulated, whereas the H3K27 methyltransferase complex component *Suz12* was downregulated in the absence of *Prdm14*. This is of particular interest, because loss of histone 3 lysine-9 dimethylation (H3K9me2) and the upregulation of histone 3 lysine-27 trimethylation (H3K27me3) fail to occur in *Prdm14*-deficient PGCs [3].

Loss of *Prdm14* also caused de-repression of the DNA methyltransferase *Dnmt3b* and its co-factor *Dnmt3l*. Importantly, genes including *Dazl*, *Tex19.1* and *Tex19.2*, as well as the Rhox gene family that are regulated by their promoter DNA methylation [12, 13], were repressed in *Prdm14* mutant ES cells, indicating that *Prdm14*-mediated suppression of DNA methyltransferases is necessary for their expression. We confirmed retention of DNA methylation in the promoter regions of these genes in *Prdm14*-null cells by bisulphite analysis (Fig 3D).

Taken together, our data suggest that loss of *Prdm14* leads to reduced expression of germline genes and induces somatic lineage priming in ES cells, despite culture in 2i conditions. Furthermore, *Prdm14* is critical for repression of DNA methyltransferases, particularly of *Dnmt3b*, to enable expression of germline genes.

### *Prdm14* maintains naive pluripotency

**Figure 4:**
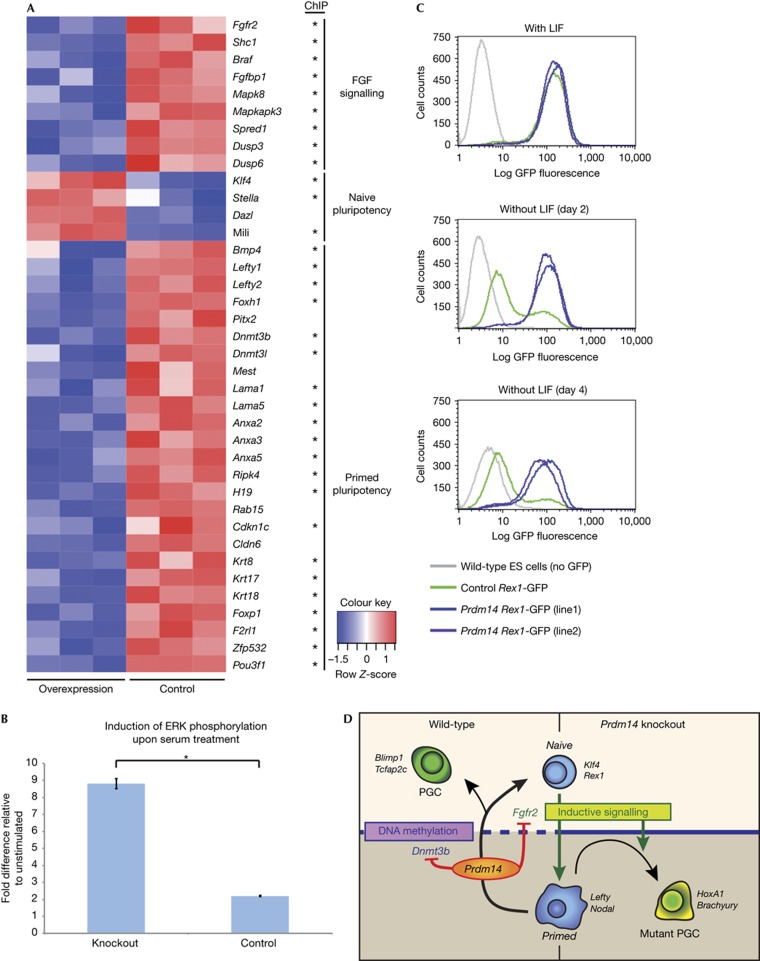
*Prdm14* represses lineage markers and renders ES cells partially LIF independent. (**A**) Heatmap of differentially expressed genes (FDR<0.01) in control versus *Prdm14* gain of function ES cells cultured in serum. Genes determined to be direct target genes by ChIPseq for PRDM14 in mouse ES cells [7] are highlighted by an asterisk. (**B**) Induction of ERK phosphorylation in control and *Prdm14*-null ES cells upon 15 min serum stimulation. Values show fold change compared to levels in unstimulated cells, as determined by quantitative ELISA assay (*n*= three independent biological repeats). Error bars show standard error of the mean and statistical significance was assessed by *t*-test (*P⩽0.05). (**C**) Flow cytometry analysis of *Rex1-*GFP levels after 2 and 4 days of LIF withdrawal in serum culture conditions in control ES cells compared to two independent ES cell lines overexpressing *Prdm14*. Data shown are representative of independent biological repeats, with 100,000 cells profiled for each cell line. (**D**) Model and summary of *Prdm14* function in ES cells and PGCs. ELISA, enzyme-linked immunosorbent assay; ERK, extracellular signal-regulated kinase; ES, embryonic stem; FGF, fibroblast growth factor; GFP, green fluorescent protein; FDR, false discovery rate; PGC, primordial germ cell; s.e.m., standard error of the mean.

The unexpected upregulation of differentiation markers in *Prdm14*-deficient ES cells in 2i conditions suggested that *Prdm14* might contribute to reducing gene expression heterogeneity in ES cells when cultured in the presence of serum [18]. We therefore examined if *Prdm14* gain of function can mimic the effects of 2i culture and reduce heterogeneity in ES cells (Fig 4A; supplementary Table S2 online). We found that genes including *Klf4, Mili, Dazl* and *Stella* were upregulated, whereas genes associated with a differentiation-primed state, such as *Cldn6*, *Otx2*, *Lefty1/2* and *Pitx2* [19], were downregulated (Fig 4A; supplementary Fig S3A,B online). In addition, heterogeneity in ES cells with respect to PE fates was also repressed upon *Prdm14* overexpression, which is consistent with a previous report [7].

ES cell priming for differentiation is mediated by fibroblast growth factor (FGF) signalling [10] and its pharmacological inhibition reduces heterogeneity [15]. Strikingly, we found that multiple components of the FGF pathway, such as *Fgfr2*, *Braf* and *Shc1*, which were shown to be direct targets of *Prdm14* [7], were downregulated when *Prdm14* levels are elevated (Fig 4A, supplementary Fig 3C online). This suggests that the reduction in expression of early differentiation genes could be a consequence of an attenuation of the FGF pathway by PRDM14. Indeed, expression of FGF signalling-induced genes, such as *Spred1* and *Dusp6* [20], was reduced upon *Prdm14* overexpression. Moreover, there are similarities between gene expression changes caused by *Prdm14* overexpression and those ascribed to direct inhibition of the FGF signalling pathway [20]. To gain further evidence that *Prdm14* acts in part through attenuation of MAPK pathway activation, we determined the induction of ERK phosphorylation by a quantitative enzyme-linked immunosorbent assay (ELISA) assay upon exposure of ES cells to serum. We found that *Prdm14*-null ES cells show a stronger induction of the pathway compared to wild-type cells (Fig 4B). These findings demonstrate that *Prdm14* limits the sensitivity of ES cells to differentiation-inducing signals.

Consistently, we also found that *Prdm14* limits the continuous FGF signalling-induced drift of ES cells out of a naive pluripotent state that is commonly observed under standard culture conditions [10], as judged by the expression of *Rex1* in ES cells. Thus, the majority of ES cells overexpressing *Prdm14* reside within the *Rex1*-high expression state that indicates naive pluripotency (supplementary Fig S3D online). We also found that the expression of key pluripotency factors, Kruppel-like factor 4 (KLF4) and NANOG, was significantly increased, with a clear reduction of cells showing low expression (supplementary Fig S3E online). Furthermore, we found that constitutive expression of *Prdm14* limits spontaneous differentiation of ES cells grown in the presence of LIF, generating mostly undifferentiated colonies with strong alkaline phosphatase staining (supplementary Fig S3F online).

We next tested if constitutive expression of *Prdm14* could maintain the pluripotent state in the absence of LIF as judged by the extent of *Rex1* expression, which discriminates between naive and differentiation-primed ES cell states [18] (Fig 4C). Within 2 days of LIF withdrawal, the majority of control cells drifted into the *Rex1-*negative state, and only a minority of them were *Rex1* positive after 4 days. In contrast, most of the *Prdm14*-overexpressing cells remained in the *Rex1* positive state after 2 and 4 days of LIF withdrawal; thereafter we observed cell death, which might be due to their inability to differentiate. We also found that while withdrawal of LIF in control cells led to a strong reduction in the expression levels of pluripotency genes, these levels were only slightly diminished in cells with constitutive expression of *Prdm14* (supplementary Fig S4A,B online).

In summary, we found that *Prdm14* represses components of the FGF signalling pathway, thereby limiting ERK activation. Consequently, genes associated with naive pluripotency are induced and genes characteristic for a differentiation-primed state are repressed in ES cells overexpressing *Prdm14*.

## CONCLUDING REMARKS

Here we provide further insights on the impact of loss of *Prdm14* function on PGC development and in ES cells. Our results indicate that the defects in *Prdm14* mutant cells arise from a lack of repression of the DNA methyltransferase machinery, and by a failure to attenuate the differentiation-inducing FGF signalling pathway. These conclusions are in agreement with other recent studies that describe the role of *Prdm14* concerning pluripotency and self-renewal of ES cells [21, 22]. PRDM14 functions appear to be particularly critical for resetting the epigenome, re-establishment of the pluripotency network and maintenance of germline fate *in vivo* (Fig 4D). While *Prdm14* is critical for PGC development and in promoting pluripotency in ES cells, there was no detectable effect of loss of *Prdm14* on pre- or peri-implantation development [3] when pluripotency is established. This might be because all traversed states during development are short lived as cells quickly transit through cell fate specification programmes. This is unlike the prolonged maintenance of pluripotency of ES cells in culture, where negative regulation of FGF signalling helps to inhibit lineage commitment. Notably, the type of lineage deregulation in the absence of *Prdm14* was context dependent, suggesting that *Prdm14* counteracts the generation of differentiation-primed states in general in ES and PGCs, and not of one specific lineage. It is striking that despite culture in 2i conditions, absence of *Prdm14* leads to upregulation of some differentiation markers in ES cells, indicating a possible additional contribution of *Prdm14* to the homogenous gene expression profile of naive pluripotency [23]. Our findings highlight a unique function for *Prdm14* in regulating signalling and epigenetic states that modulate the balance between pluripotency and differentiation.

## METHODS

**Generation of *Prdm14* heterozygous mice and genotyping.** C57BL/6 ES cells heterozygous for *Prdm14* were obtained from the EUCOMM repository and injected into E3.5 C57BL/6 host blastocysts. Knockout embryos were obtained by heterozygous crosses. All husbandry and experiments involving mice were carried out according to the local ethics committee and were performed in a facility designated by the Home Office.

**Single-cell cDNA library preparation.** For isolation of PGCs, embryos at the 4–6 somites stage from timed heterozygous crosses of mice bearing a *Stella-GFP* reporter were dissected and single PGCs from individual genotyped embryos sorted using a MoFlo MLS high-speed flow sorter (Beckman Coulter). Generation of cDNAs and subsequent amplification were performed as described by Tang *et al* [24].

**ES cell derivation, culture and manipulation.** ES cells were derived in 2i conditions as detailed previously [14]. ES cells were cultured either in gelatin-coated dishes under standard culture conditions with serum or in fibronectin-coated dishes under 2i culture conditions, both as described previously [6]. For generation of stable *Prdm14* overexpressing ES cell lines, the transcriptional *Rex1*-GFPd2 (*Zfp42*-GFPd2) reporter ES cell line [25] was used as parental line. Quantitation of induction of ERK phosphorylation was performed using an ELISA kit (R&D Systems). Bisulphite sequencing was performed as previously described [12].

**Quantitative real-time PCR and global gene expression analysis.** RNA was extracted using the RNeasy Kit (Qiagen) and cDNA was synthesized using SuperScript III reverse transcriptase (Life Technologies). All quantitative PCR runs were performed and analysed as detailed previously [6]. For genome-wide expression analysis, 1 μg of total RNA was used for labelling and hybridization to Mouse WG-6 Expression BeadChips (Illumina). Differential expression was calculated using LIMMA v3.6.0, *P*-values were corrected for multiple testing and probes with *P*<0.01 were deemed significant. Microarray data are available from the GEO depository under the accession number GSE45509.

**Whole-mount immunostaining.** Embryos from timed heterozygous matings were dissected and processed for immunostaining as described previously [26]. Images were acquired using a confocal microscope (Olympus) and analysed with Volocity software (Perkin Elmer). Primary antibodies used were as follows: anti-GFP (Nacalai Tesque, GF090R), anti-BLIMP1 (eBiosciences, 14-5963-80); anti-OCT-3/4 (BD Transduction Laboratories, 611203); anti-phosphoERK1/2 (Thr202/Tyr204) (Cell Signalling Technology, #9101); anti-FGFR2 (Sigma-Aldrich, SAB1403815).

Supplementary information is available at EMBO *reports* online (http://www.emboreports.org).

## Supplementary Material

Supplementary Information

Supplementary Table 1

Supplementary Table 2

Review Process File
