# Supplementary material for: Prdm14 promotes germline fate and naive pluripotency by repressing FGF signalling and DNA methylation
Source: EMBO Rep. 2013 May 14;14(7):629–37. doi: 10.1038/embor.2013.67 (PMC3701237; doi:10.1038/embor.2013.67)
Supplement: Supplementary Information [file embor201367s1.pdf]

Supplementary Information

Prdm14 promotes germline fate and naïve pluripotency by repressing FGF signaling and DNA methylation

Nils Grabole, Julia Tischler, Jamie A. Hackett, Shinseog Kim, Fuchou Tang[#], Harry G. Leitch, Erna Magnúsdóttir and M. Azim Surani*

Supplementary data (Figure S1 – S4)

Supplementary tables (Table S1 and S2, provided separately as Excel files)

Supplementary material and methods

Supplementary references

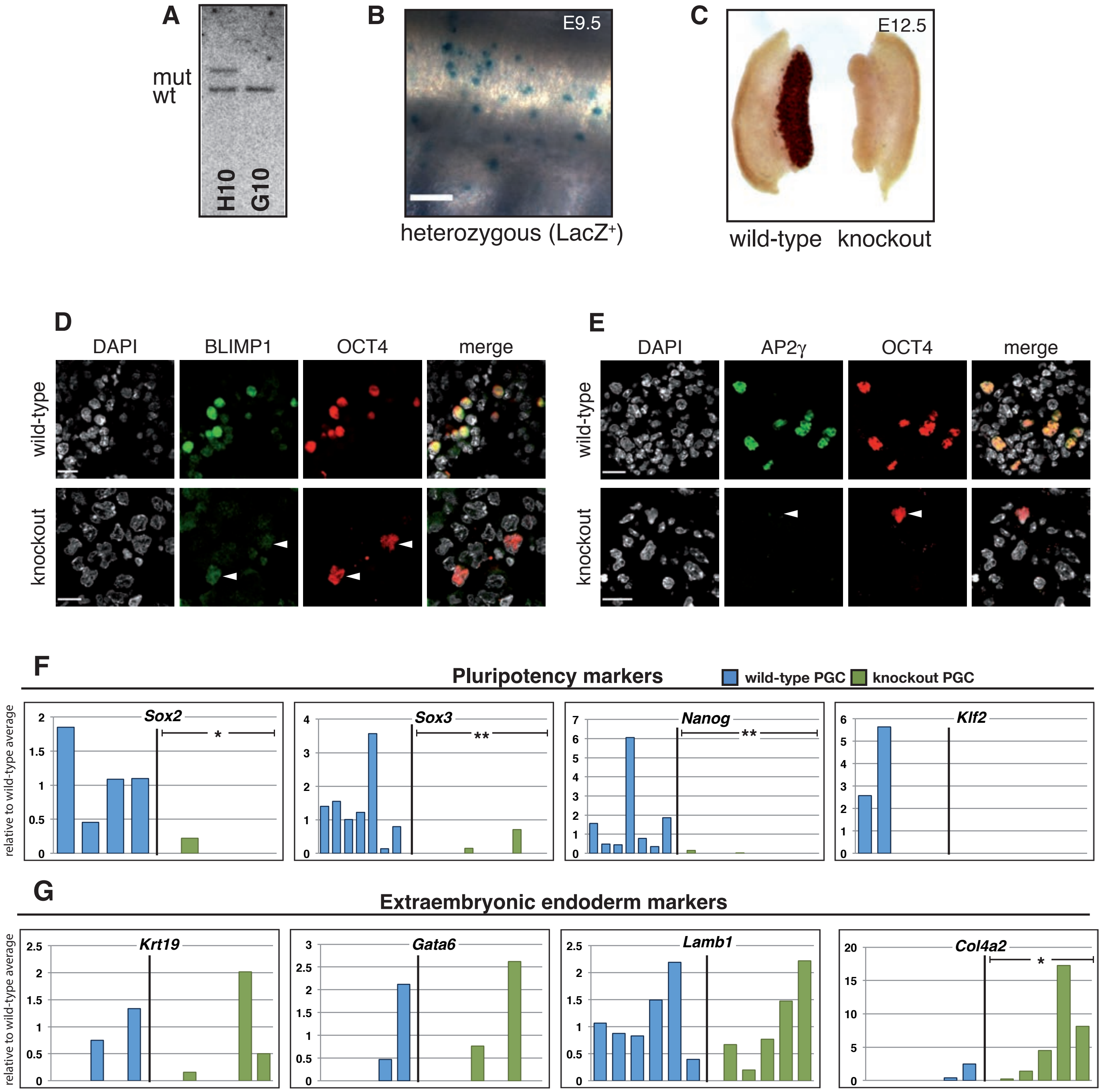

Supplementary figure S1. *Prdm14*-deficient PGCs show aberrant expression profiles on the transcript and protein level but do not acquire extraembryonic endoderm fate (related to Figure 1)

(A) Southern blot analysis of genomic DNA extracted from targeted ES cell clones, indicating correct targeting of the *Prdm14* locus in the 'H10' clone. (B) Detection of PGCs by LacZ staining in *Prdm14* heterozygous mice at E9.5. (C) Alkaline phosphatase stain confirming complete loss of PGCs prior to colonization of the gonads in *Prdm14* mutant mice at E12.5. Immunostainings for (D) BLIMP1 at E8.5 and (E) AP2γ at E10.5 on cryosections of *Prdm14*-deficient embryos or littermates. PGCs are identified by staining for OCT4 (arrow heads) Scale bar = 20 µm. (F,G) Analysis of expression of pluripotency genes (F) and extra-embryonic endoderm fate markers (G) as described in Figure 1.

A

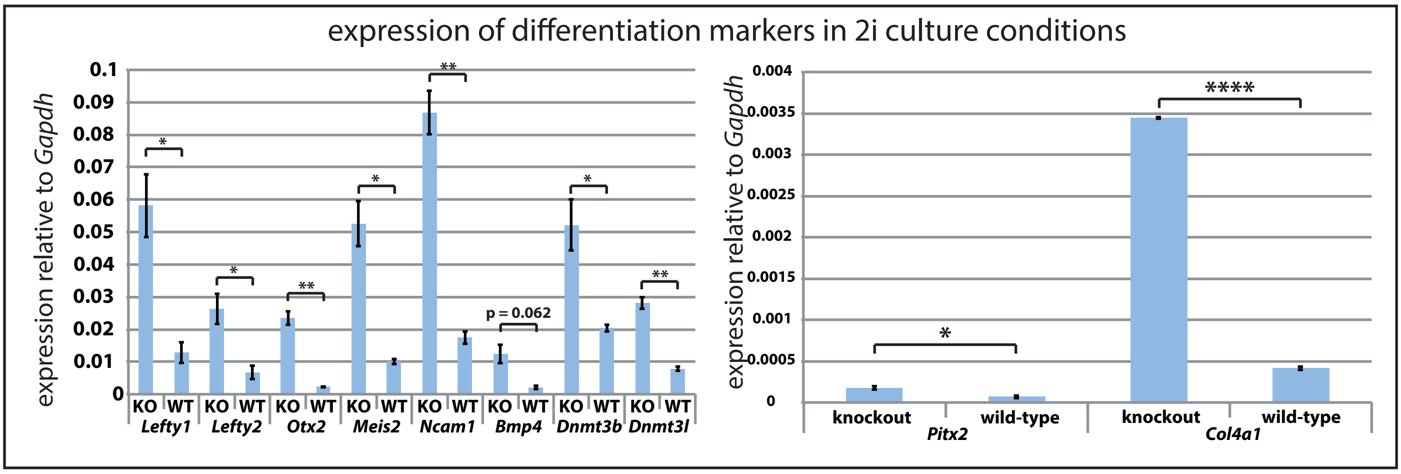

B

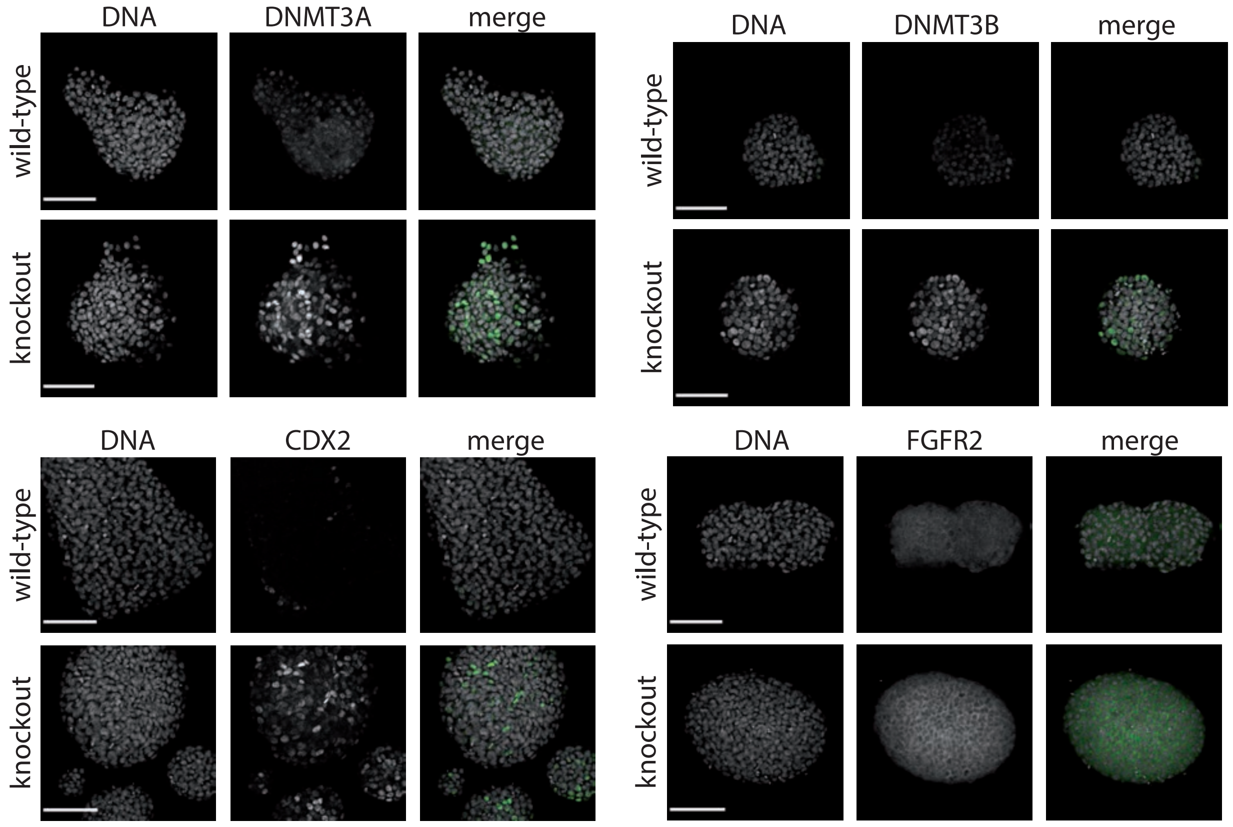

Supplementary figure S2. Expression of differentiation markers in wild-type and *Prdm14* knockout ES cells in 2i culture conditions (related to Figure 3)

A) Determination of expression levels relative to *Gapdh* by qPCR of differentiation genes detected to be differentially expressed between wild-type and *Prdm14* knockout ES cells in 2i culture (Figure 3C). Error bars indicate standard error of the mean of biological triplicates. Statistical significance was determined by a t-test (* $p \leq 0.05$; ** $p \leq 0.01$; **** $p \leq 0.0001$). B) Immunostaining of ES cells in 2i conditions showing expression heterogeneity and increased protein levels in knockout cells (Scale bar = 100 μm).

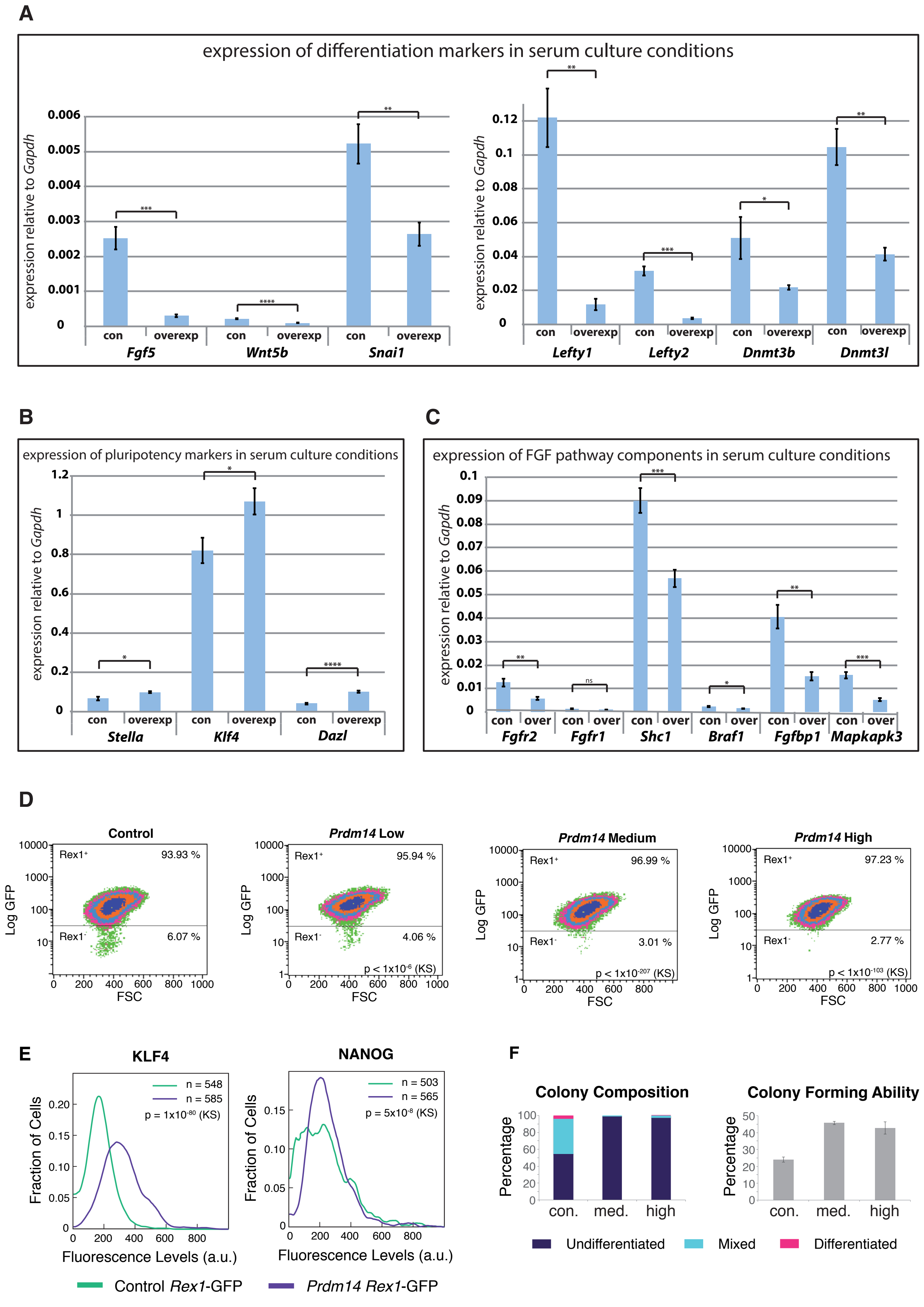

A
expression of differentiation markers in serum culture conditions
expression relative to Gapdh
0.006
0.005
0.004
0.003
0.002
0.001
0
con
overexp
Fgf5
Wnt5b
Snai1
0.12
0.1
0.08
0.06
0.04
0.02
Lefty1
Lefty2
Dnmt3b
Dnmt3l
B
expression of pluripotency markers in serum culture conditions
1.2
1
0.8
0.6
0.4
0.2
Stella
Klf4
Dazl
C
expression of FGF pathway components in serum culture conditions
0.1
0.09
0.08
0.07
0.06
0.05
0.04
0.03
0.02
0.01
over
ns
Fgfr2
Fgfr1
Shc1
Braf1
Fgfbp1
Mapkapk3
D
Control
Prdm14 Low
Prdm14 Medium
Prdm14 High
Log GFP
FSC
Rex1+
Rex1-
93.93 %
6.07 %
95.94 %
4.06 %
p < 1x10-6 (KS)
96.99 %
3.01 %
p < 1x10-207 (KS)
97.23 %
2.77 %
p < 1x10-103 (KS)
E
KLF4
n = 548
n = 585
p = 1x10-80 (KS)
NANOG
n = 503
n = 565
p = 5x10-8 (KS)
Fraction of Cells
Fluorescence Levels (a.u.)
Control Rex1-GFP
Prdm14 Rex1-GFP
F
Colony Composition
Colony Forming Ability
Percentage
con.
med.
high
Undifferentiated
Mixed
Differentiated

Supplementary figure S3. *Prdm14* overexpression represses differentiation genes and FGF pathway components and reduces spontaneous differentiation of ES cells (related to Figure 4)

(A-C) qPCR validation of transcript levels for genes associated with early differentiation (A), pluripotency (B) or FGF pathway components (C) that were found to be differentially expressed by microarray analysis, as shown in Figure 3. Data shown represent the mean of six biological replicates and is relative to *Gapdh*. Error bars represent the standard error of the mean; statistical significance was determined by a t-test (* $p \leq 0.05$, ** $p \leq 0.01$, *** $p \leq 0.001$, **** $p \leq 0.0001$). (D) Flow cytometry analysis showing *Rex1*-GFP distributions of ES cells with different expression levels of *Prdm14*. Percentages indicate fractions of cells within the *Rex1*-positive and *Rex1*-negative expression regimes. A Kolmogorov-Smirnov (KS) test was used to determine the statistical significance of differences to wild-type cells. (E) Distributions of KLF4 and NANOG levels in *Rex1*-GFP control ES cells or ES cells stably expressing *Prdm14*. A KS-test was used to determine the statistical significance of differences in protein level distributions. (F) Composition of ES cell colonies was analysed by an alkaline phosphatase (AP) assay twelve days after plating. Colonies were scored as follows: ≥90% AP-positive cells, undifferentiated; ≥ 50% AP-positive cells, mixed; ≤50% AP-positive cells, differentiated. Plating efficiencies of control ES cells or ES cells overexpressing medium and high levels of *Prdm14* were analysed by determing the percentage of cells having formed a colony twelve days after plating in standard culture conditions.

A

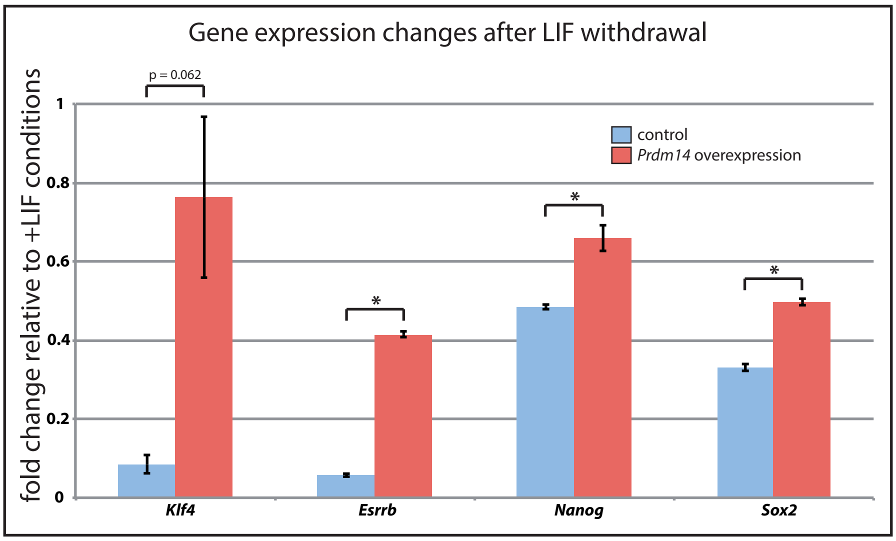

B

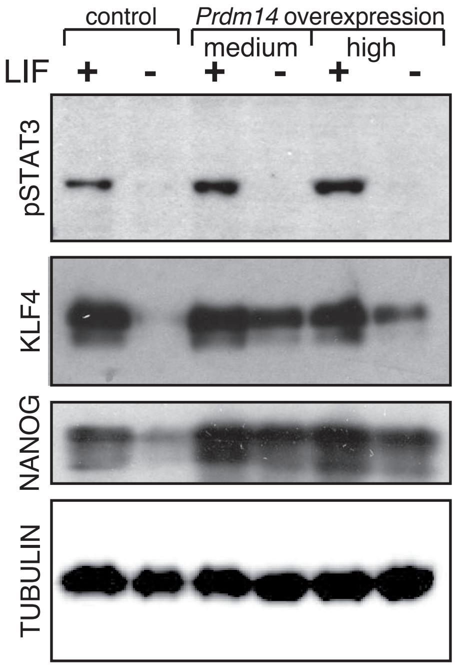

Supplementary figure S4. *Prdm14* overexpression reduces LIF dependence of embryonic stem cells (related to Figure 4)

(A) Expression levels of pluripotency factors determined by qPCR in control ES cells or ES cells overexpressing *Prdm14* after two days of LIF withdrawal in serum culture conditions. Expression is relative to cells grown in self-renewal conditions with serum and LIF and was normalized to *Gapdh*. Error bars represent the standard error of the mean and statistical significance was assessed by a t-test (* $p \leq 0.05$). (B) Protein levels in *Prdm14* overexpression and control cell lines after two days of LIF withdrawal. α-TUBULIN was used as loading control.

Supplemental methods

Mouse strains and embryo dissections:

For whole-mount immunofluorescence, *Blimp1*-GFP mice [1], maintained on a mixed CBA and C57BL/6 background, or *Stella*-GFP-BAC mice [2], maintained on a C57BL/6 genetic background were used. *Prdm14*$^{tm1e(EUCOMM)wtsi/+}$ mice (*Prdm14* $^{-/+}$) were maintained on a pure C57BL/6 background unless crossed to *Stella*-GFP-BAC mice to label PGCs by GFP for FACS sorting. Embryos were staged according to Downs and Davies [3] before somitogenesis, while somite numbers were counted thereafter. Embryos were dissected out of uterus and decidua in PBS under a Leica Wild 3M dissecting microscope (Leica Microsystems) and washed in PBS with 0.2% polyvinylpyrrolidone (Sigma-Aldrich) before further processing by dissociation or fixation. Images were acquired on a Zeiss SV11 dissecting microscope equipped with a colour digital camera (Jenoptik ProgRes C14) using Openlab image software (PerkinElmer Improvision).

Histological methods:

For tissue-nonspecific alkaline phosphatase (TNAP) staining, dissected gonads from E12.5 embryos were fixed in 4 % paraformaldehyde (PFA) for 2 hours at 4 °C, washed in PBS, incubated in 70 % ethanol for 1 hour at 4 °C and then washed again in water. The embryos were then transferred into staining solution (3 ml water, 0.5 ml of 5 mg/ml 1,5-naphthalenedisulfonate Fast Red, 0.5 ml of 1mg/ml sodium 1-naphthyl phosphate, 0.5 ml of 0.6 % magnesium chloride and 0.5 ml of sodium 5,5-diethylbarbiturate; all from Sigma-Aldrich). The staining was carried out at room temperature and monitored through a dissection microscope. The staining procedure was stopped when the stain was sufficiently developed (approximately after 10 to 20 minutes) by washing the gonads in water to avoid background stain. Stained gonads were cleared in 70 % glycerol overnight at room temperature and mounted onto object slides for imaging.

For β-Galactosidase staining, dissected embryos were fixed between 10 and 30 minutes, depending on the developmental stage, in 0.4 % glutaraldehyde in 0.1 M phosphate buffer (pH 7.4) with 2 mM magnesium chloride and 5 mM EGTA. The embryos were then washed four times for 15 minutes at room temperature in washing solution (0.1 M phosphate buffer with

2 mM magnesium chloride, 0.01 % sodium deoxycholate and 0.02 % NP-40). For staining, 5 mM potassium ferricyanide, 5 mM potassium ferrocyanide (both from Sigma-Aldrich) and 1 mg/ml X-gal (Life Technologies) were added to the washing solution. Embryos were incubated in this staining solution at 37 °C until the stain was satisfactory. Staining was stopped by washing twice with washing solution and twice with PBS for 5 minutes. Stained embryos were post-fixed in 4 % PFA for 1 hour at room temperature and washed with PBS three times for 5 minutes. To visualize stained PGCs, the embryos were cleared in 70 % glycerol overnight and then mounted on an object slide.

To prepare cryosections of mouse gonads or posterior embryo fragments, tissues were fixed at 4 °C in 4 % PFA for 2 hours and were then washed three times for 15 minutes in PBS. For cryoprotection, tissues were passaged through a series of 10 %, 15 % and 20 % sucrose in PBS, for 30 minutes each at room temperature, before placing the samples in a 1:1 mix of PBS/20 % sucrose and OCT compound (VWR) for at least 30 minutes. The samples were then embedded in OCT compound in moulds, frozen on dry ice and stored at -80 °C until further processing by cryotomy. Sections of 8-12 µm thickness were cut with a Leica cryostat and collected onto Superfrost Plus slides (VWR). After air-drying, the slides were either washed in PBS and immediately used for immunocytochemistry or stored at 4 °C. To immunostain the cryosections, the specimen was permeabilized in 0.25 % Triton X-100 in PBS (PBS-T) for 30 minutes at room temperature washed twice in PBS for 5 minutes and then blocked in blocking buffer containing 2 % normal donkey serum (Jackson ImmunoResearch), 0.1 % BSA and 0.01 % Tween20 (Sigma-Aldrich) for 1 hour at room temperature. The specimen was incubated with primary antibodies (anti-BLIMP1 (1:50, eBiosciences, rat mAb 14-5963-80); anti-OCT-3/4 (1:200, BD Transduction Laboratories mouse mAb 611203); anti-AP2g (1:200, Santa Cruz Biotechnology, rabbit pAb sc-8977)) diluted in blocking buffer overnight at 4 °C in a humidified chamber. The following day, the samples were washed three times for 15 minutes in PBS-T and then incubated with the appropriate secondary antibodies (Molecular Probes, Life Technologies) diluted in blocking buffer containing 100 ng/ml DAPI for 1 hour at room temperature. Finally, the samples were washed twice in PBS-T and once in PBS for 15 minutes at room temperature. The object slides were mounted with Vectashield and the slides stored at 4 °C until imaging. Images were acquired as described above as described in the Whole-mount immunostaining section.

Immunofluorescence

For immunofluorescence of ES cells, cells were grown on fibronectin-coated glass-coverslips. Cells were washed twice in 1xPBS, fixed in 4% PFA in PBS for 10-15 minutes at room temperature, followed by three washes in 1xPBS. Cells were subsequently permeabilized in 0.1% Tween-20 in PBS for 10-15 minutes at room temperature before incubation/blocking in blocking buffer (5% FBS, 0.1% Tween-20 in PBS) for 1 hour at room temperature. Cells were then incubated with primary antibody (anti-KLF4 (1:167, goat IgG, AF3158, R&D Systems); anti-NANOG (1:167, rabbit IgG, Abcam); anti-GFP (1:500, rat IgG)) diluted in blocking buffer overnight at 4 °C in a humidified chamber in the dark. The following day, cells were washed three times in blocking buffer before incubation with secondary antibodies (Molecular Probes, Life Technologies) diluted 1:500 in blocking buffer for 1 hour at room temperature. Finally, cells were washed three times in 1x PBS at room temperature, before mounting coverslips in Vectashield plus DAPI on microscope slides. Slides were sealed with nailpolish and stored at 4 °C until imaging. Images were acquired on a Leica SP5 confocal microscope. Fluorescence levels were quantified using Python Software.

Colony formation assay

For colony formation assays, ES cells were plated at a dilution of 600 cells per well onto gelatine-coated 6-well culture dishes (Nunc), with 3 wells per genotype, in standard serum/LIF (GMEM/10%FBS/1000U LIF/ml) conditions. Culture media was replaced on day 1 and day 8 after seeding cells, with media containing 500U/ml of LIF. Colonies were stained on day 12 using an Alkaline Phosphatase Kit (Sigma-Aldrich) to assess colony composition and plating efficiencies.

Western and Southern Blot

Whole cell extracts were prepared by lysis in RIPA buffer on ice for 30 minutes and subjected to SDS-PAGE. The membrane was probed with the following antibodies: anti-tubulin (1:5000, Sigma-Aldrich, mouse mAb T9026); anti-phosphoSTAT3(Y705) (1:1000, Cell Signalling

Technology, rabbit pAb #9131), anti-NANOG (1:1000, Abcam, rabbit pAb ab80892), anit-KLF4 (1:1000, R&D Systems, goat pAb AF3158).

For Southern blot genomic DNA extracted from ES cell clones G10 or H10 was digested overnight with AatI or AflII restriction enzymes, precipitated with ethanol, separated on 0.8 % agarose gels and transferred onto a Hybond-N^+ membrane (Amersham) by capillary transfer. For detection of the different *Prdm14* alleles, a $^{32}\alpha$P dCTP-labelled probe was generated using the Prime-a-gene labelling kit (Promega) and purified with Probe Quant G-50 micro columns (Amersham). The sequences of the primers used to generate the southern probe were: forward: CACCATTCCAGTTCCTCTTTG and reverse: TGCAGGTATATTTGCCTCAAT. Following overnight-incubation with the probe, the membrane was washed and exposed to a screen for 2 to 3 days before scanning on a phosphoimager (Fujifilm).

Supplemental references:

1. Ohinata Y *et al* (2005) Blimp1 is a critical determinant of the germ cell lineage in mice. *Nature* **436:** 207-213
2. Payer B, Lopes SMCdS, Barton SC, Lee C, Saitou M, Surani MA (2006) Generation of stella-GFP transgenic mice: a novel tool to study germ cell development. *Genesis* **44:** 75-83
3. Downs KM, Davies TJ (1993) Staging of gastrulating mouse embryos by morphological landmarks in the dissecting microscope. *Development* **118:** 1255-1266
