# Supplementary material for: Prdm14 promotes germline fate and naive pluripotency by repressing FGF signalling and DNA methylation
Source: EMBO Rep. 2013 May 14;14(7):629–37. doi: 10.1038/embor.2013.67 (PMC3701237; doi:10.1038/embor.2013.67)
Supplement: Review Process File [file embor201367s4.pdf]

EMBO
reports

Manuscript EMBOR-2012-36894

Prdm14 promotes germline fate and naïve pluripotency by repressing FGF signaling and DNA methylation

Nils Grabole, Julia Tischler, Jamie A. Hackett, Shinseog Kim, Fuchou Tang, Harry G. Leitch, Erna Magnusdottir and M. Azim Surani

Corresponding author: M. Azim Surani, University of Cambridge

Review timeline:

	Submission date:	22 November 2012
	Accepted:	18 April 2013

Transaction Report:

No Peer Review Process File is available with this article, as the authors have chosen not to make the review process public in this case.

© European Molecular Biology Organization
